# Supplementary material for: A wireless battery-free eye modulation patch for high myopia therapy
Source: Nat Commun. 2024 Feb 26;15:1766. doi: 10.1038/s41467-024-46049-6 (PMC10897479; doi:10.1038/s41467-024-46049-6)
Supplement: Supplementary file 1 — Supplementary Information [file 41467_2024_46049_MOESM1_ESM.pdf]

Supplementary Materials for

A wireless battery-free eye modulation patch for high myopia therapy

Tianyan Zhong[1], Hangjin Yi[2,3], Jiacheng Gou[4], Jie Li[3], Miao Liu[3], Xing Gao[3], Sizhu Chen[3], Hongye Guan[1], Shan Liang[1], Qianxiong He[2], Rui Lin1, Zhihe Long[5], Yue Wang[4], Chuang Shi[4], Yang Zhan[6], Yan Zhang[1], Lili Xing[1], Jie Zhong[2,3*], Xinyu Xue[1*]

[1]School of Physics, University of Electronic Science and Technology of China, Chengdu 611731, China.
[2]School of Medicine, University of Electronic Science and Technology of China, Chengdu 610054, China.
[3]Department of Ophthalmology, Sichuan Provincial People's Hospital, University of Electronic Science and Technology of China, Chengdu, China.
[4]School of Information and Communication Engineering, University of Electronic Science and Technology of China, Chengdu 611731, China.
[5]Department of Mechanical Engineering, City University of Hong Kong, Hong Kong SAR 999077, China.
[6]Brain Cognition and Brain Disease Institute, Shenzhen Institutes of Advanced Technology, Chinese Academy of Sciences, Shenzhen 518055, China.

*Corresponding author. Email: zhongjie@med.uestc.edu.cn; xuexinyu@uestc.edu.cn

This PDF file includes:

Supplementary Figures 1-34
Supplementary Note 1

Supplementary Fig. 1. Depiction of patch treatment in high myopia progression. 4
Supplementary Note 1. 5
Supplementary Fig. 2. Wireless eye modulation high myopia patch. 6
Supplementary Fig. 3. Illustration of the circuit board of ultrasonic.. 7
Supplementary Fig. 4. Schematic illustrations the output performance of the lead zirconate titanate (PZT) transducer. 8
Supplementary Fig. 5. The indicator utilized to assist in positioning during implantation procedures. 9
Supplementary Fig. 6. Illustration of the μ-LEDs of the myopia patch during electrolysis and collagen cross-linking (SCXL). 10
Supplementary Fig. 7. Propagation processes of ultrasound sound pressure within the rabbit eye. 11
Supplementary Fig. 8. Optical image of the interdigitated electrode. 12
Supplementary Fig. 9. Electrolysis characteristic. 13
Supplementary Fig. 10. The cyclic voltammetry curve in three cycles. 14
Supplementary Fig. 11. Optos image reveals the implant position of the myopia patch in porcine eye. 15
Supplementary Fig. 12. The infrared thermal image of the micro-actuator. 16
Supplementary Fig. 13. The infrared thermal image of the lead zirconate titanate (PZT) transducer. 17
Supplementary Fig. 14. Spectral characteristic. 18
Supplementary Fig. 15. Characterization of drug release by microneedles. 19
Supplementary Fig. 16. The fluorescence intensity of porcine sclera tissues during 0-30 min of riboflavin drug diffusion. 20
Supplementary Fig. 17. Characterization of optical and mechanical properties. 21
Supplementary Fig. 18. Hematoxylin and eosin (H&E) staining of the porcine sclera. 22
Supplementary Fig. 19. Implantation and characterization of myopia patch. 23
Supplementary Fig. 20. Ultrasonography (A-B Scan) revealing pre- and post-treatment variations in axial length... 24
Supplementary Fig. 21. Hematoxylin and eosin (H&E) staining of rabbit sclera section at 14 days post-treatment. 25
Supplementary Fig. 22. Structure and electron microscopy of control and scleral collagen cross-linking (SCXL) rabbit ocular tissue. 26
Supplementary Fig. 23. Monitoring of intraocular pressure (IOP) changes over a 22-day period. 27
Supplementary Fig. 24. Immunohistological analysis right eye (OD) and left eye (OS) of rabbit. 28
Supplementary Fig. 25. Toluidine blue-stained semithin sections of the control and scleral collagen cross-linking (SCXL) eye of an adult rabbit. 29
Supplementary Fig. 26. Tunel assay detection of apoptosis control group and scleral collagen cross-linking (SCXL) group. 30
Supplementary Fig. 27. Preoperative and postoperative fundus photography and fluorescein angiography (FFA) comparisons. 31
Supplementary Fig. 28. Daily body temperature and body weight of rabbits. 32
Supplementary Fig. 29. Optical imaging of the right eye (OD) and left eye (OS) 22 days post-surgery.. 33

Supplementary Fig. 30. Schematic illustrations of the assembly of the myopia patch. 34
Supplementary Fig. 31. The mold of myopia patch. 35
Supplementary Fig. 32. Characterization of the flexible polydimethylsiloxane/polystyrene-block-polybutadiene-block-polystyrene (PDMS/SBS) membrane. 36
Supplementary Fig. 33. Positive mold of the microneedle array.. 37
Supplementary Fig. 34. Attachment technique and location for the patch. 38

Supplementary Fig. 1.

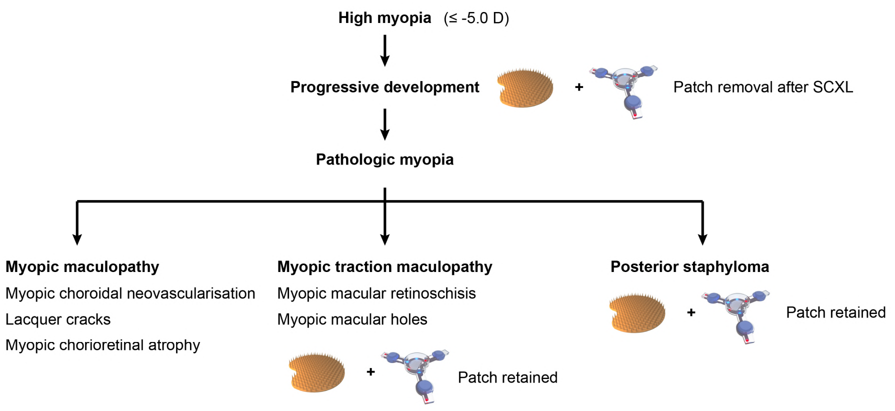

Depiction of patch treatment in high myopia progression. Progressive high myopia is highly likely to evolve into pathological myopia.

Supplementary Note 1.

Pressure in the pump chamber.

To quantitatively predict the pressure in the micro-actuator and drug reservoir, a theoretical model is established for the whole micro-actuator system. Ideal gas law of the pump chamber gives

$$P(V + V_0) = nRT \qquad \text{(Eq S1)}$$

where P is the pressure, V_0 and V are the initial volume change of air inside the micro-actuator, respectively, n is the number of moles of air, R is the ideal gas constant, and T the temperature.

From Faraday's laws of electrolysis

$$Q = nZF \qquad \text{(Eq S2)}$$

where Q is the total charge passed, Z is the number of electron transfers for the product, and F is the Faraday constant.

it can be known that the number of moles of gas molecules

$$n = \frac{3it}{4F} \qquad \text{(Eq S3)}$$

From Eq S1, the volume of the micro-actuator V can be relater i by

$$V = \frac{3it}{4FP}RT \qquad \text{(Eq S4)}$$

The volume of deformation V generated by the chamber can be equivalently represented as the volume of a dome, which can be calculated as

$$V = \pi h \frac{(h^2+3a^2)}{6} \qquad \text{(Eq S5)}$$

where h is the height of the members deformation, and a is the radius of the chamber.

The volume of a micro-actuator undergoes change when gas pressure is applied. The formula for the Bulk modulus (k) can be expressed as

$$k = \frac{E}{3(1-2\nu)} \qquad \text{(Eq S6)}$$

$$k = -V_0 \frac{\partial P}{\partial V} \qquad \text{(Eq S7)}$$

where k is Bulk modulus, E is Young's modulus and ν is Poisson ration.

According to Eq S4, Eq S5, Eq S6 and Eq S7, the functional relationship between maximum displacement of flexible membrane (h) and t can be obtained as

$$EF\pi^2 h^2 (3a^2 + h^2)^2 = 81 iRT\pi l a^2 (1 - 2\nu)t \qquad \text{(Eq S8)}$$

Supplementary Fig. 2.

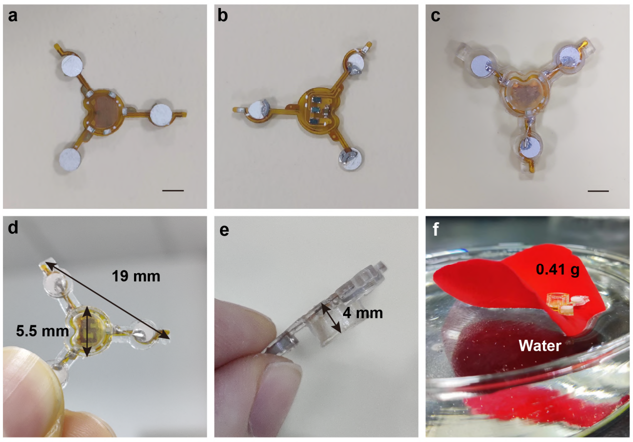

Wireless eye modulation high myopia patch. a, b Flexible micro-fabricated circuit design. Scale bar, 3 mm. **c-e** The flexible and transparent elastomer polydimethylsiloxane (PDMS) encapsulates the system. Scale bar, 3 mm. **f** The whole system weight 0.41 g.

Supplementary Fig. 3.

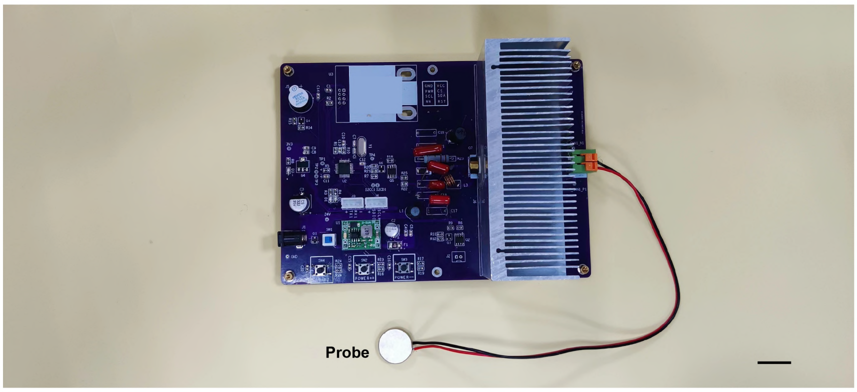

Illustration of the circuit board of ultrasonic. The ultrasonic transmitter consists of a modulation circuit and an ultrasonic probe. Scale bra, 20 mm.

Supplementary Fig. 4.

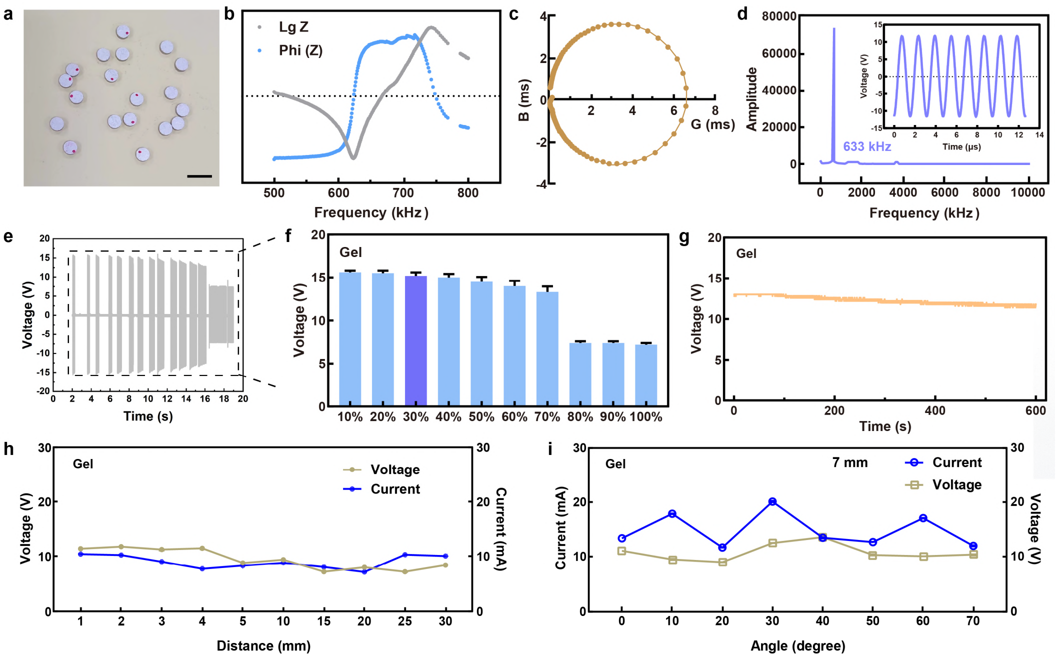

Schematic illustrations the output performance of the lead zirconate titanate (PZT) transducer. a Optical image of the size of the PZT transducer. Scale bar, 5 mm. **b** Illustrate the resonant frequency of the PZT. **c** Admittance circle diagram. **d** The Fourier transform of the output signal indicates that the frequency is 633 kHz. Insert is the output voltage of PZT transducer. **e, f** The outputs characteristics of PZT transducer at various duty cycles. Experiment study with 30% duty cycle. **g** Stable performance at 30% duty cycle of the PZT transducer. **h** Output performance of the PZT transducer at different distances in gel. **i** Output performance of the PZT transducer at different angles. Data are expressed as mean ± SD, with each experiment independently replicated 8 times (n=8). Source data are provided as a Source Data file.

Supplementary Fig. 5.

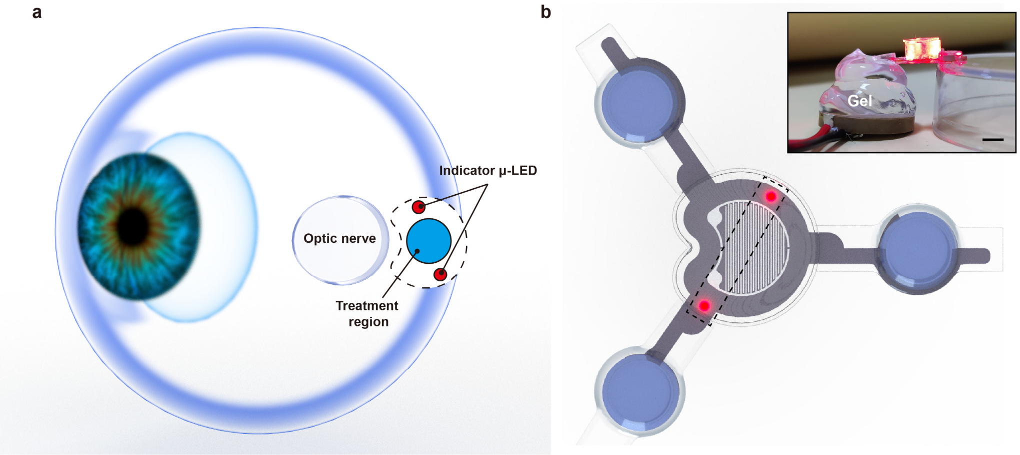

The indicator utilized to assist in positioning during implantation procedures. a Red μ-LEDs symmetrically distributed in the center of the system span both sides of the treatment area to aid in positioning during implantation. **b** The distribution of red μ-LEDs on the myopia patch. The inset: an optical image depicting the side view of the location indicator in action. Scale bar, 5 mm.

Supplementary Fig. 6.

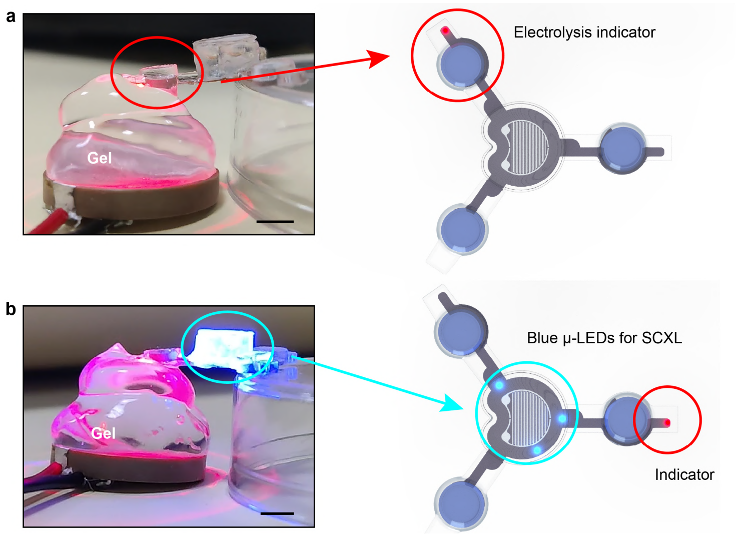

Illustration of the μ-LEDs of the myopia patch during electrolysis and sclera collagen cross-linking (SCXL). a An optical image depicting the side view of the electrolysis indicator in action. Right inset: a diagram illustrating the positioning of the red μ-LED indicator during electrolysis. Scale bar, 5 mm. **b** An optical image depicting the side view of the μ-LEDs and indicator of SCXL in action. Right inset: a diagram illustrating the positioning of the blue μ-LEDs and red μ-LED indicator during SCXL. Scale bar, 5 mm.

Supplementary Fig. 7.

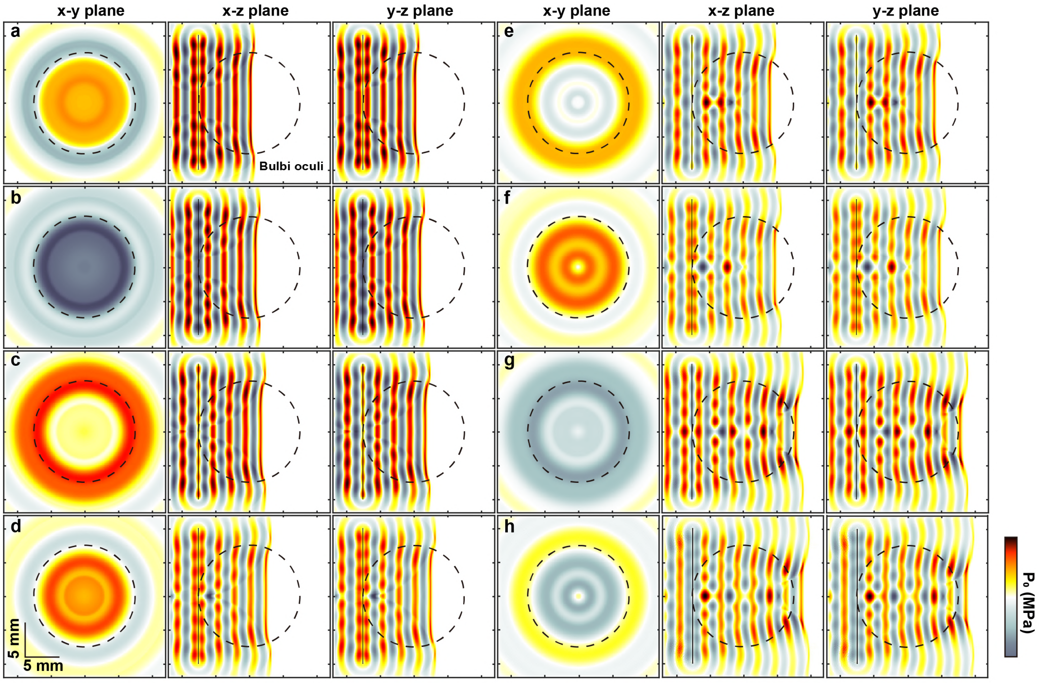

Propagation processes of ultrasound sound pressure within the rabbit eye. a-h, Simulation results indicate a minor attenuation of acoustic pressure (P_0 ~ 0.6 MPa) through gel, vitreous, and sclera, maintaining perpendicular alignment to the transducer surface. The effective operational area spans roughly a 10 mm diameter, with a minimum separation over 15 mm between adjacent transducers, surpassing the rabbit eyeball's diameter, thus effectively preventing unintended activation of other functionalities.

Supplementary Fig. 8.

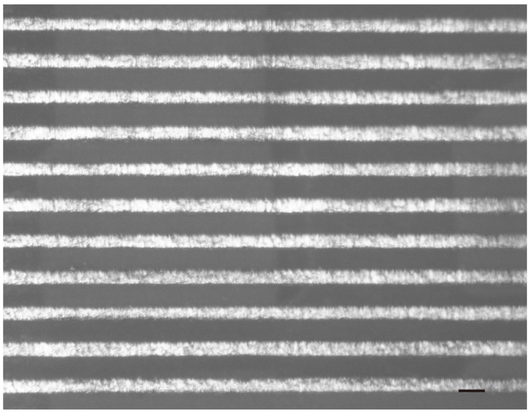

Optical image of the interdigitated electrode. Cu/Au interdigitated electrodes feature a precise width of 60 µm. Scale bra, 100 µm.

Supplementary Fig. 9.

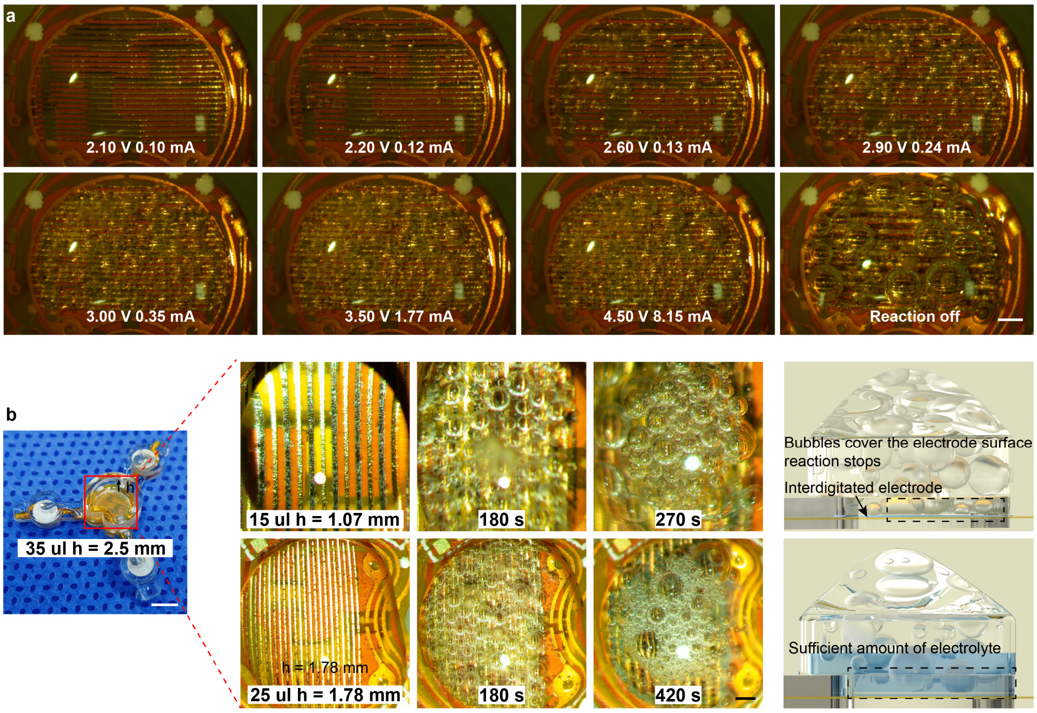

Electrolysis characteristic. a The optical photographs of bubble generation at different voltage. **b** The left image displays a liquid chamber with a capacity of 35 µL, fully utilized. Scale bar, 3 mm. The right image shows the chamber at its minimum operational capacity of 25 µL, indicating that electrolysis below this threshold leads to premature cessation due to bubble accumulation over the electrode, caused by an insufficient volume of solution. Scale bar, 500 µm.

Supplementary Fig. 10.

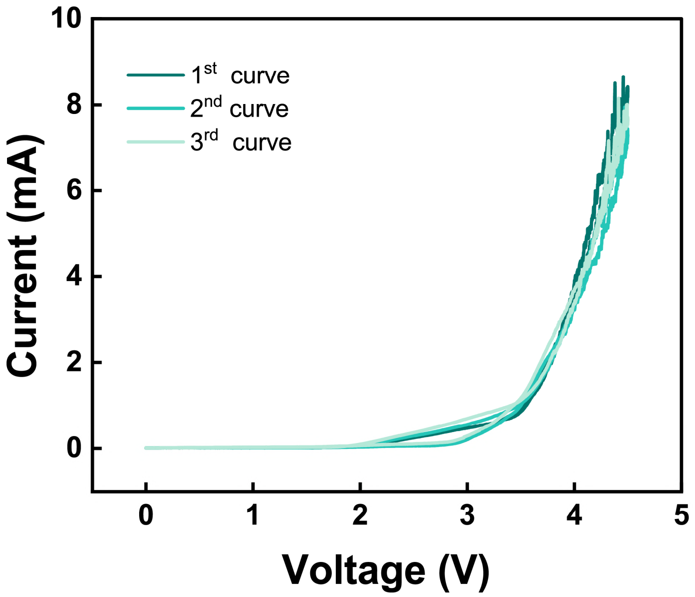

The cyclic voltammetry curve in three cycles. Under three cycles, the electrolytic capacity remains consistent, demonstrating the device's multi-cycle usability. Source data are provided as a Source Data file.

Supplementary Fig. 11.

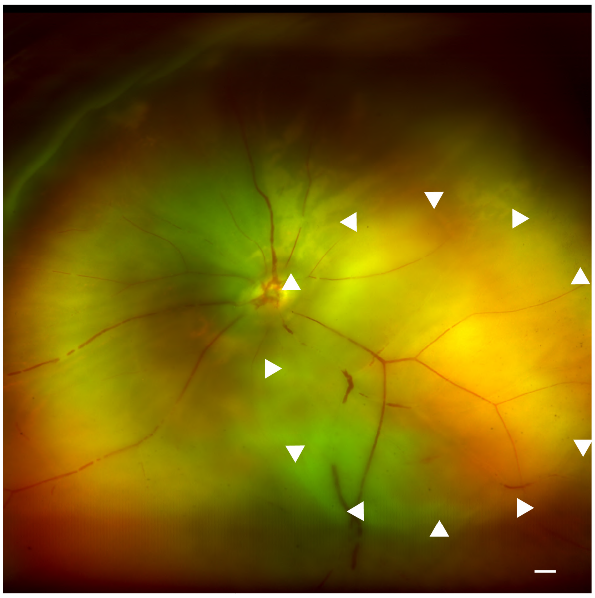

Optos image reveals the implant position of the myopia patch in porcine eye. A significant foreign entity near the optic nerve leads to a deformation in the fundus region. Scale bar, 1 mm.

Supplementary Fig. 12.

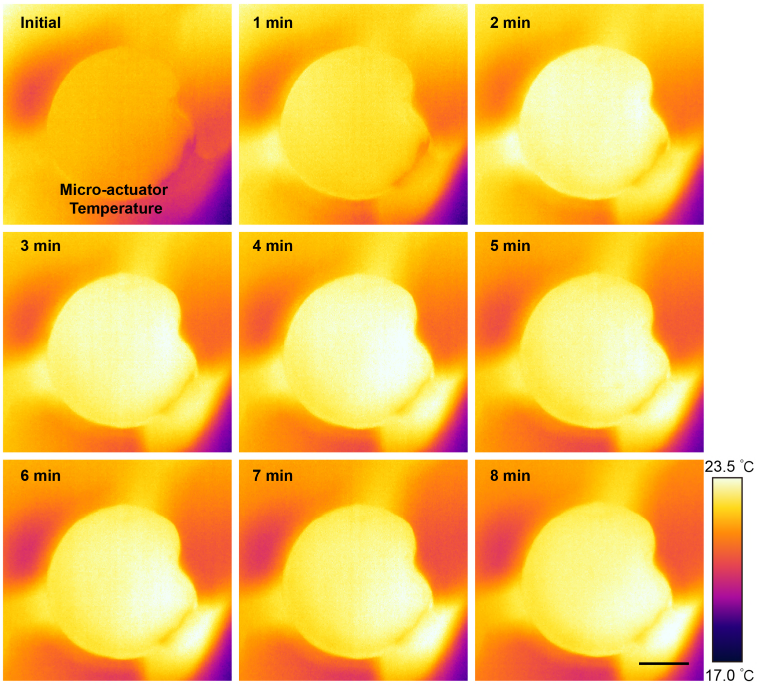

The infrared thermal image of the micro-actuator. The temperature of the micro-actuator increases slightly from 22.2ºC to 23.5ºC within 6 min. Scale bar, 2 mm.

Supplementary Fig. 13.

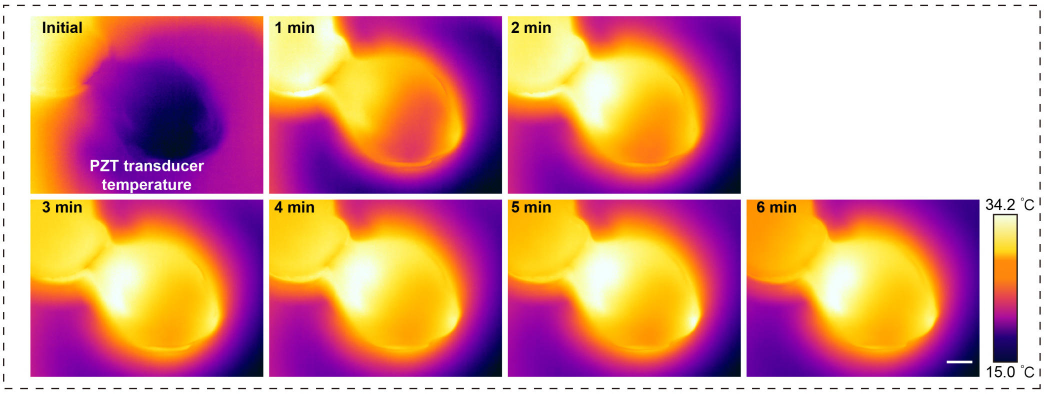

The infrared thermal image of the lead zirconate titanate (PZT) transducer. The temperature of the PZT transducer increases from 21.9ºC to 34.6ºC within 6 min. Scale bar, 1 mm.

Supplementary Fig. 14.

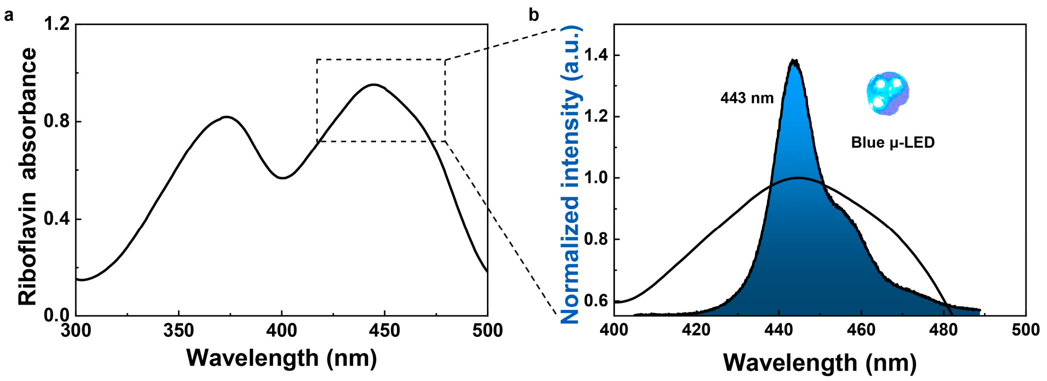

Spectral characteristic. a The absorption spectrum of riboflavin at 300-500 nm wavelength. **b** The blue μ-LED emission spectrum and the normalized intensity (a.u.) of riboflavin fluorescence. Source data are provided as a Source Data file.

Supplementary Fig. 15.

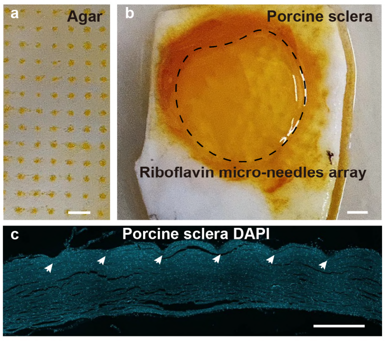

Characterization of drug release by microneedles. a Uniform array structure of the microneedle in the agarose. Scale bar, 800 µm. **b** The array structure of the microneedle in the porcine sclera. Scale bar, 1 mm. **c** Cross-sectional image of nuclei [4′,6-diamidino-2-phenylindole (DAPI)] stained porcine sclera tissue at the site of microneedle array penetration. Scale bar, 800 µm.

Supplementary Fig. 16.

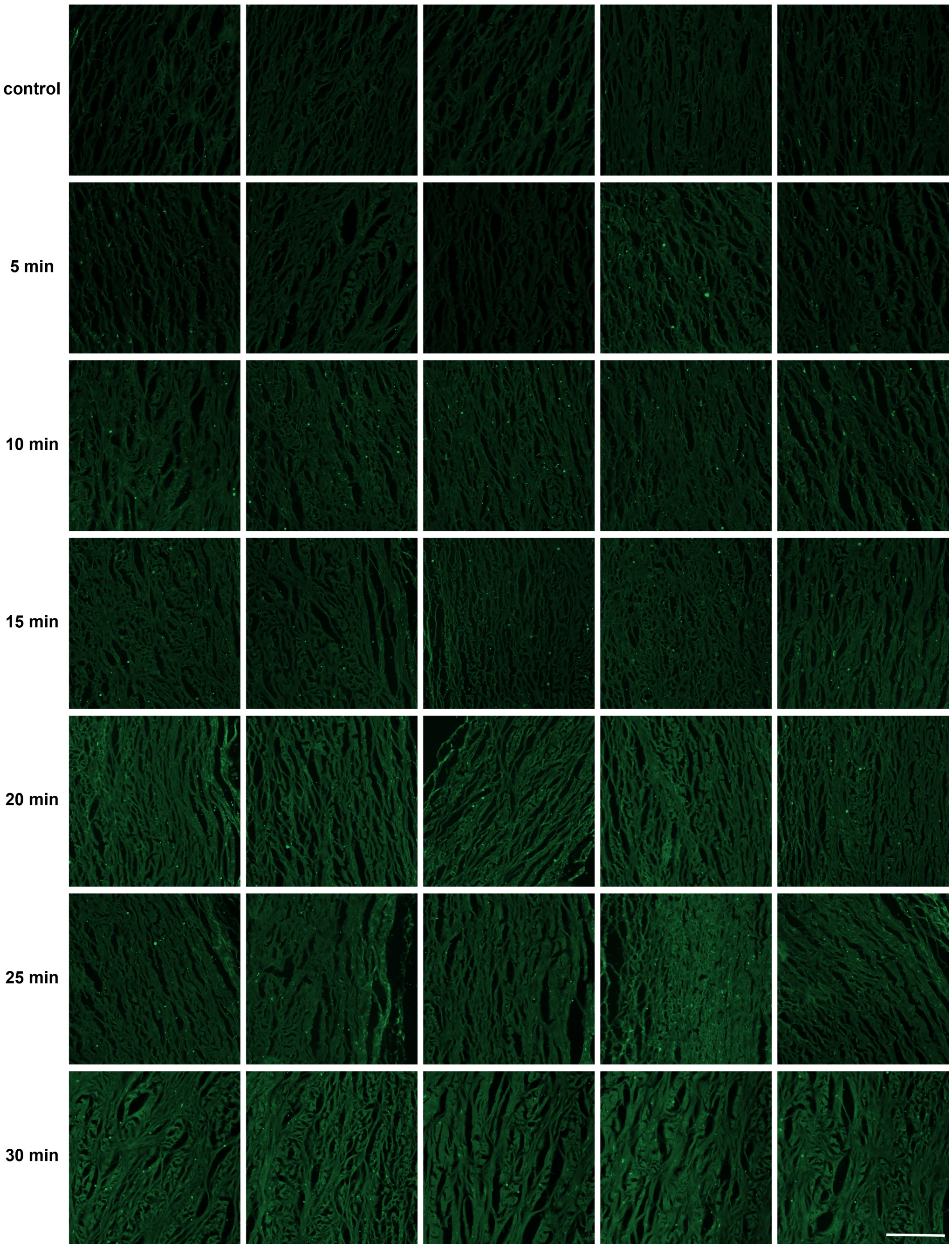

The fluorescence intensity of porcine sclera tissues during 0-30 min of riboflavin drug diffusion. Fluorescence intensity within scleral tissue progressively escalates, signifying the sustained delivery of riboflavin into the sclera. Scale bar: 200 μm.

Supplementary Fig. 17.

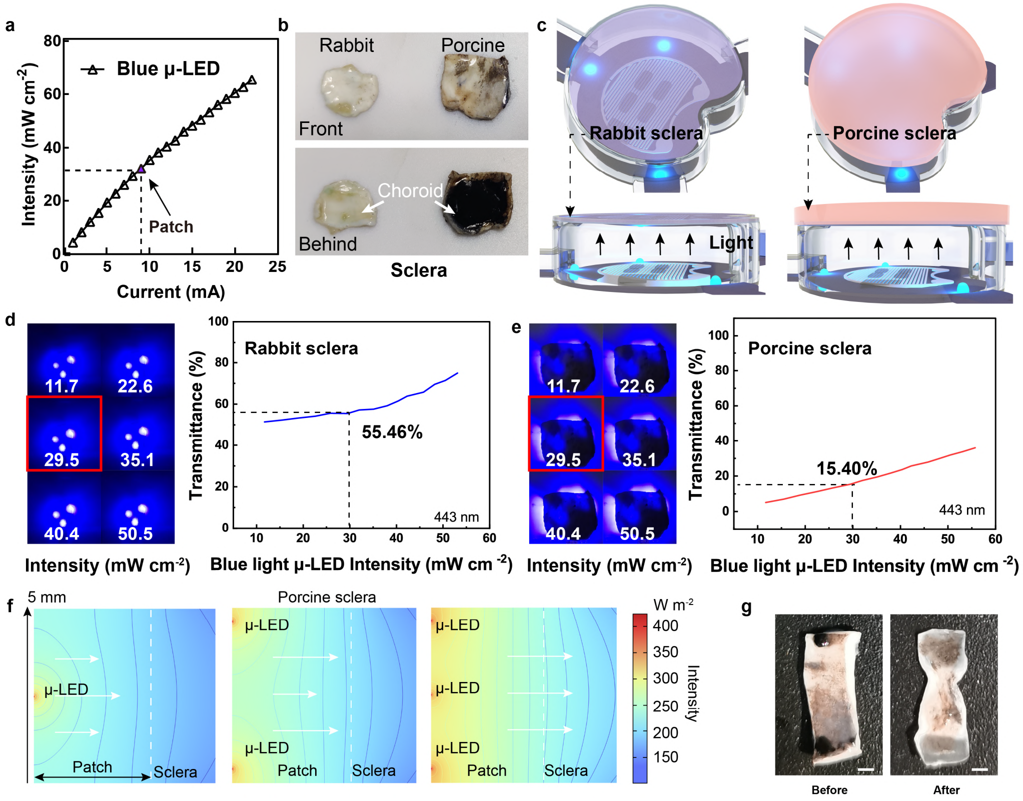

Characterization of optical and mechanical properties. a Intensity of blue μ-LED at varying current output. The data is presented as the mean of three repeated independently (n = 3). **b** Optical images of rabbit and porcine sclera. **c** Comparative diagram of light penetration in rabbit and porcine sclera: highlighting the higher pigment content in porcine sclera's choroid, greater light absorption, and reduced light transmission to the fundus compared to rabbit sclera. Light transmittance of **(d)** rabbit sclera and **(e)** porcine sclera under varying light intensity. **f** Simulation analysis: μ-LED arrangement and intensity distribution impact on porcine scleral tissue. **g** The morphological changes in the sclera before and after stretching for Young's modulus testing. Scale bar, 1 mm. Source data are provided as a Source Data file.

Supplementary Fig. 18.

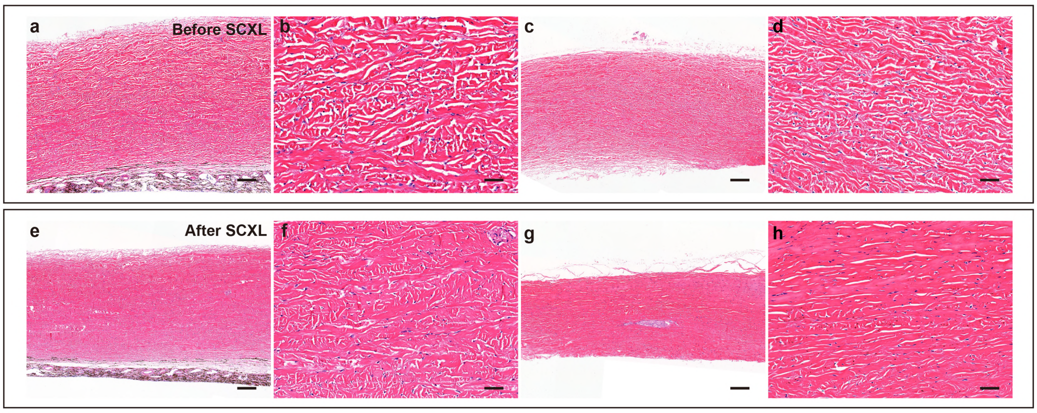

Hematoxylin and eosin (H&E) staining of the porcine sclera. a-d H&E staining of the sclera before SCXL. Scale bar in (**a, c**): 200 µm; scale bar in (**b, d**): 50 µm. **e-h** H&E staining of the sclera after collagen cross-linking. Scale bar in (**e, g**): 200 µm; scale bar in (**f, h**): 50 µm.

Supplementary Fig. 19.

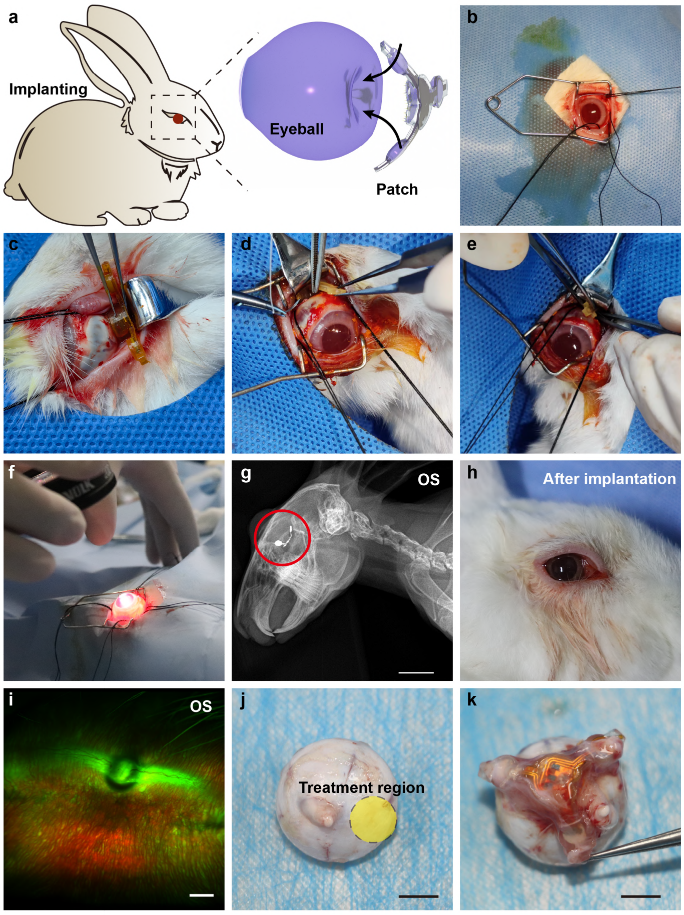

Implantation and characterization of myopia patch. a-e Implantation of a wireless eye modulation myopia patch in the right eye of a rabbit. Immobilization of the eye using sutures and a speculum, which allows for controlled protrusion of the eye. **f** Confirmation of implant location with a binocular indirect ophthalmoscope. **g** X-ray image of the rabbit left eye. Scale bar, 20 mm. **h** Optical image of the rabbit eye post-implantation of myopia patch. **i** Optos image of the left eye. Scale bar, 3 mm. **j, k** Myopia patch implantation adjacent to optic nerve in the fundus, corresponding to the macula. Scale bar, 5 mm.

Supplementary Fig. 20.

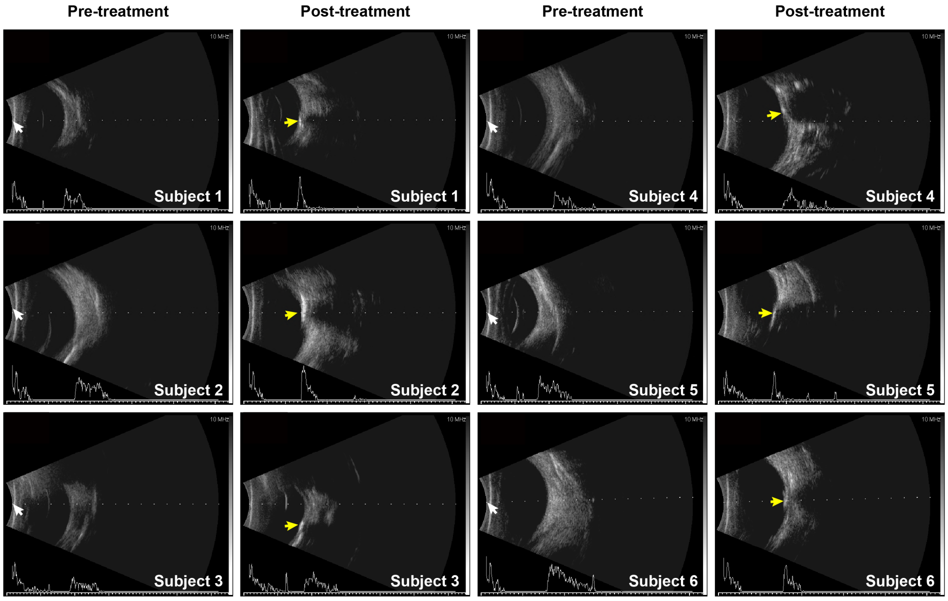

Ultrasonography (A-B Scan) revealing pre- and post-treatment variations in axial length (AXL). A-B Scan illustrates changes in AXL, demonstrating an average reduction of around 1217 µm over 6 minutes (n=6 eyes).

Supplementary Fig. 21.

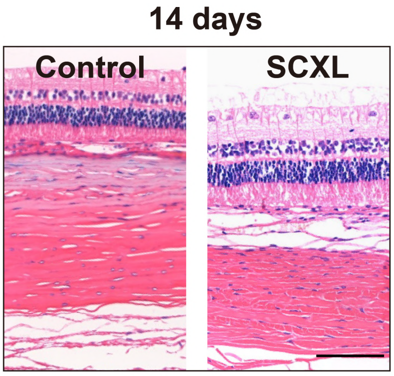

Hematoxylin and eosin (H&E) staining of rabbit sclera section at 14 days post-treatment. In the control group, H&E stained sections show loosely arranged collagen fibers, while in the cross-linking group, the collagen fibers are tightly aligned. Scale bar: 100 μm.

Supplementary Fig. 22.

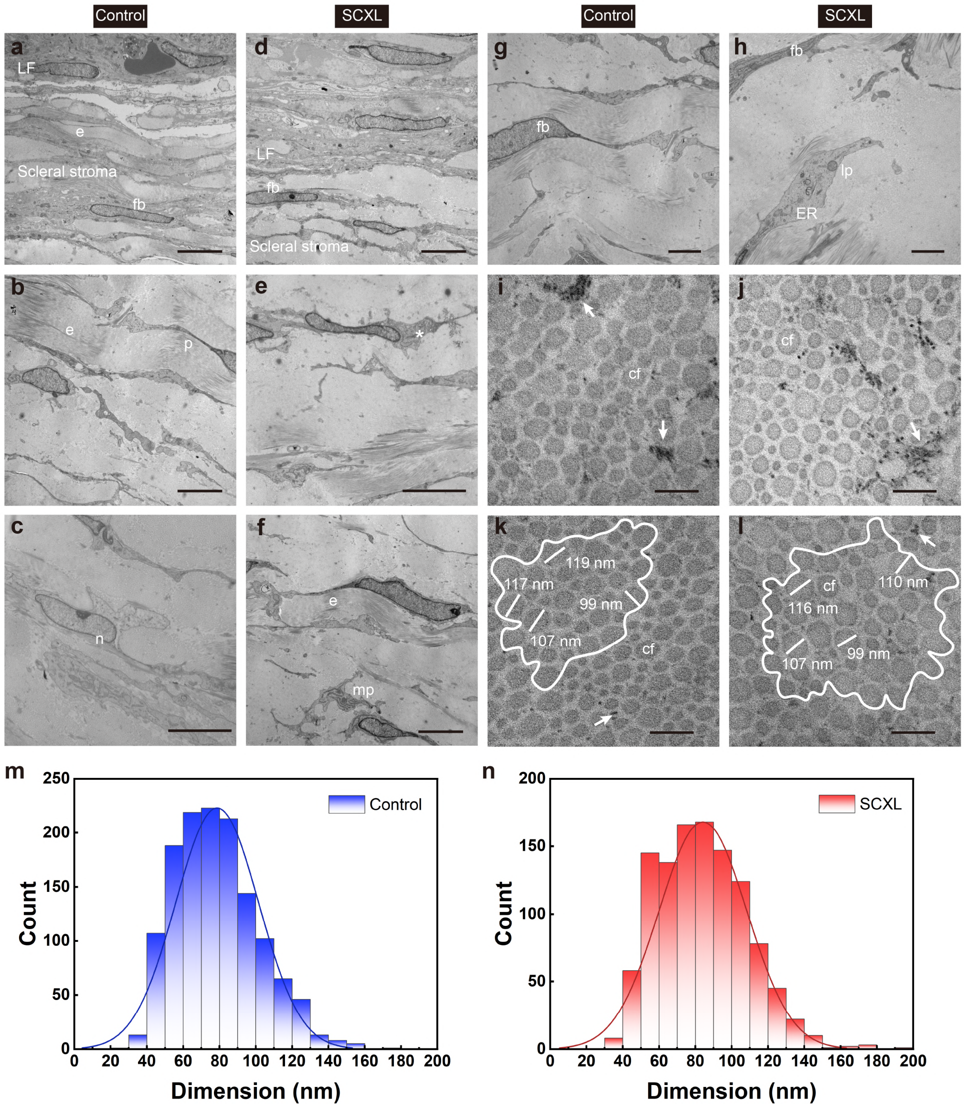

Structure and electron microscopy of control and scleral collagen cross-linking (SCXL) rabbit ocular tissue. a-c Lamina fusca (LF) and scleral stroma (Sc) in Control cases featuring elastic fibers (e) and fibroblasts (fb), with fibroblast cell bodies oriented parallel to the eyeball surface. Elliptical nuclei (n) and thin cytoplasmic processes (p) in scleral fibroblasts. Scale bar: 5 µm. **d-f** Lamina fusca (LF) and scleral stroma (Sc) post-SXCL intervention, displaying macrophage-like cells (mp) in the stroma and fibroblasts (fb) with thickened processes (*). Scale bar: 5 µm. **g-h** High magnification of fibroblasts, showing thick processes in fb post-SXCL, rich in endoplasmatic reticulum (ER) and primary lysosomes (lp). Scale bar: 200 nm. **i-l** High magnification of a frontal section of a collagen fibril bundle with collagen fibrils (cf) and elastic fibers (arrow), comparing pre- and post-SXCL. Scale bar: 200 nm. **m, n** Bar graph measuring the diameter of single collagen fibrils (m, n), indicating no diameter difference between control and post-SXCL intervention. Scale bar: 200 nm. Source data are provided as a Source Data file.

Supplementary Fig. 23.

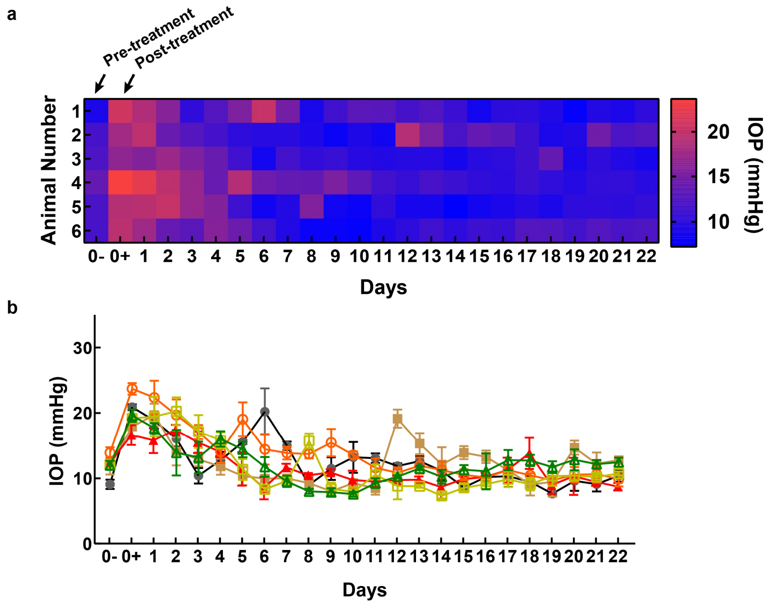

Monitoring of intraocular pressure (IOP) changes over a 22-day period: (0-) OD before surgery, (0+) OD after surgery. a Continuous monitoring over 22 days post-surgery indicated that the IOP in six rabbits nearly reverted to preoperative levels by the third day. This outcome is presented as the mean of five readings. **b** The daily variations in IOP for each rabbit were presented as mean values ± SD, with six subjects (n=6) included in the measurement. Source data are provided as a Source Data file.

Supplementary Fig. 24.

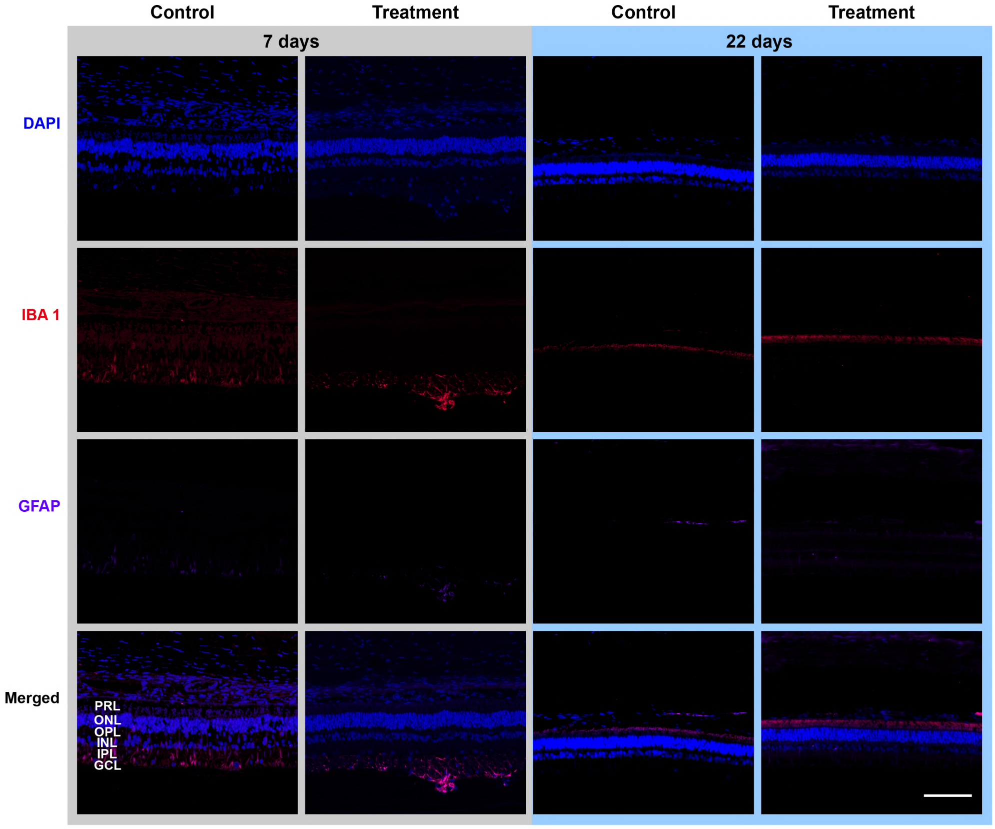

Immunohistological analysis right eye (OD) and left eye (OS) of rabbit. Visualization of cell types in the scleral collagen cross-linking (SCXL) eye and control eye of a rabbit through double immunohistochemical labeling. Double labeling of glial fibrillary acidic protein (GFAP) (purple) as an astrocytic marker and Iba-1 (red) as a microglia/macrophage marker. Resting microglia cells with tiny cell processes located in the inner plexiform layer (IPL) and ganglion cell layer (GCL). Astrocytes identified through GFAP immunonegativity. Well-preserved layered structure of the retina, with 4′,6-diamidino-2-phenylindole (DAPI) labeled cell nuclei (blue). The layers include the ganglion cell layer (GCL), inner plexiform layer (IPL), inner nuclear layer (INL), outer plexiform layer (OPL), outer nuclear layer (ONL), and photoreceptor layer (PRL). Scale bar, 100 µm.

Supplementary Fig. 25.

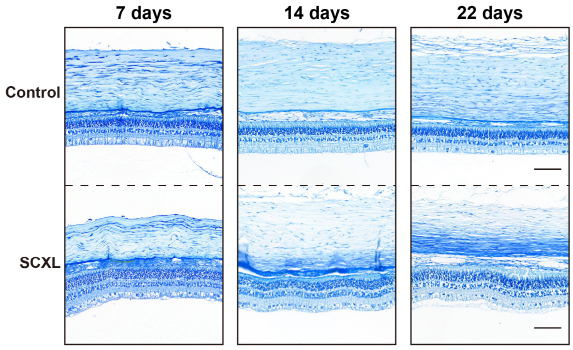

Toluidine blue-stained semithin sections of the control and scleral collagen cross-linking (SCXL) eye of an adult rabbit. The staining results reveal the typical structures of the retina, choroid, and sclera, with no observed degradation or structural loss. Scale bar, 100 μm.

Supplementary Fig. 26.

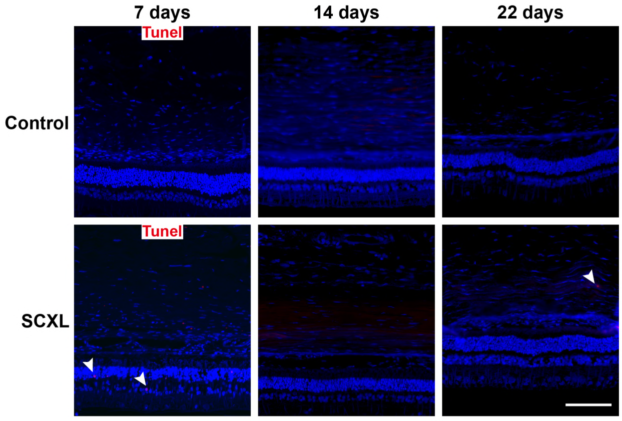

Tunel assay detection of apoptosis control group and scleral collagen cross-linking (SCXL) group. Positive signal detection indicated by an arrowhead, displaying both red (TMR stained) and blue (DAPI stained) fluorescence. Scale bar, 100 μm.

Supplementary Fig. 27.

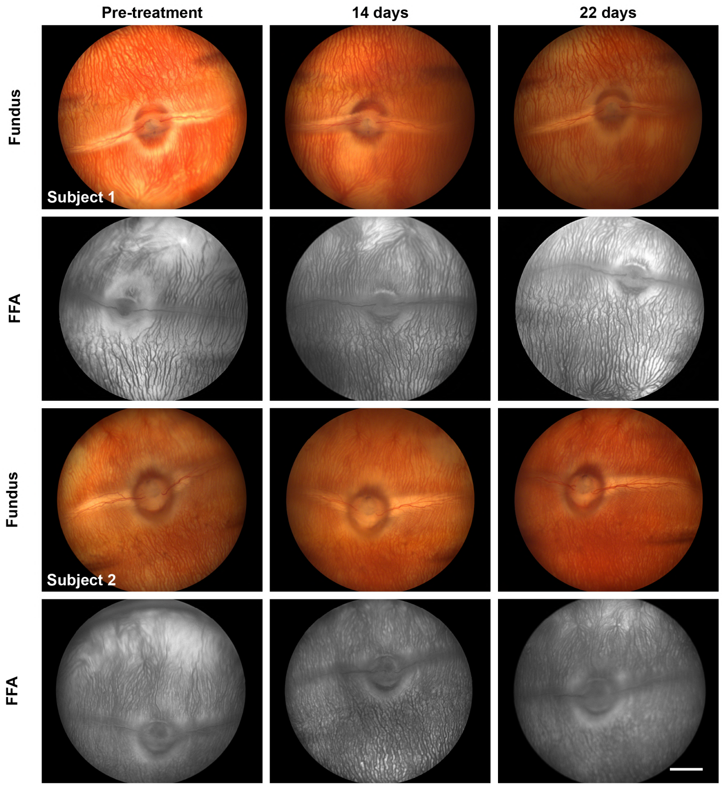

Preoperative and postoperative fundus photography and fluorescein angiography (FFA) comparisons. No severe abnormal fundus or FFA changes observed at 14 and 22 days postoperatively compared to preoperative fundus. Absence of fluorescent leakage spots in posterior retina based on FFA images. Scale bar, 2 mm.

Supplementary Fig. 28.

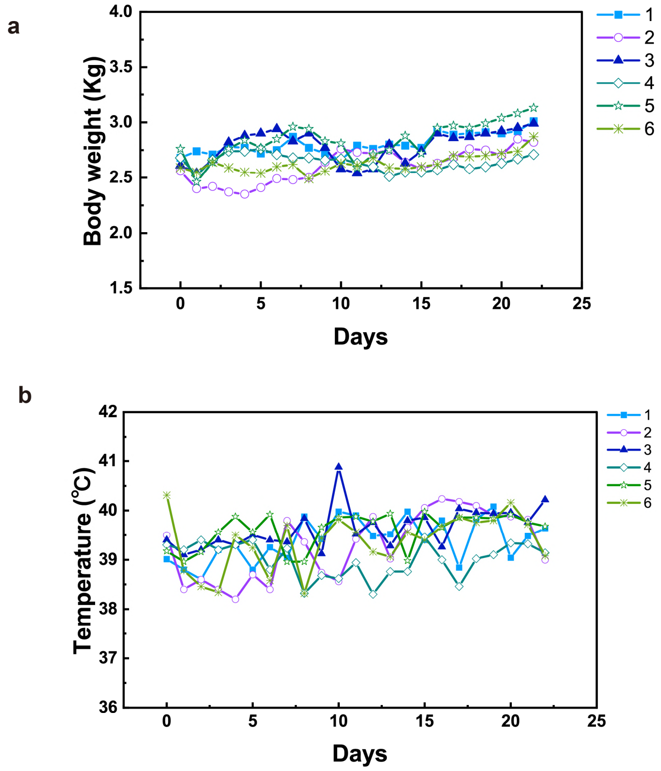

Daily body temperature and body weight of rabbits. a Body weight and **b** temperature fluctuations of rabbits over a 22-day period (n = 6 rabbits). Source data are provided as a Source Data file.

Supplementary Fig. 29.

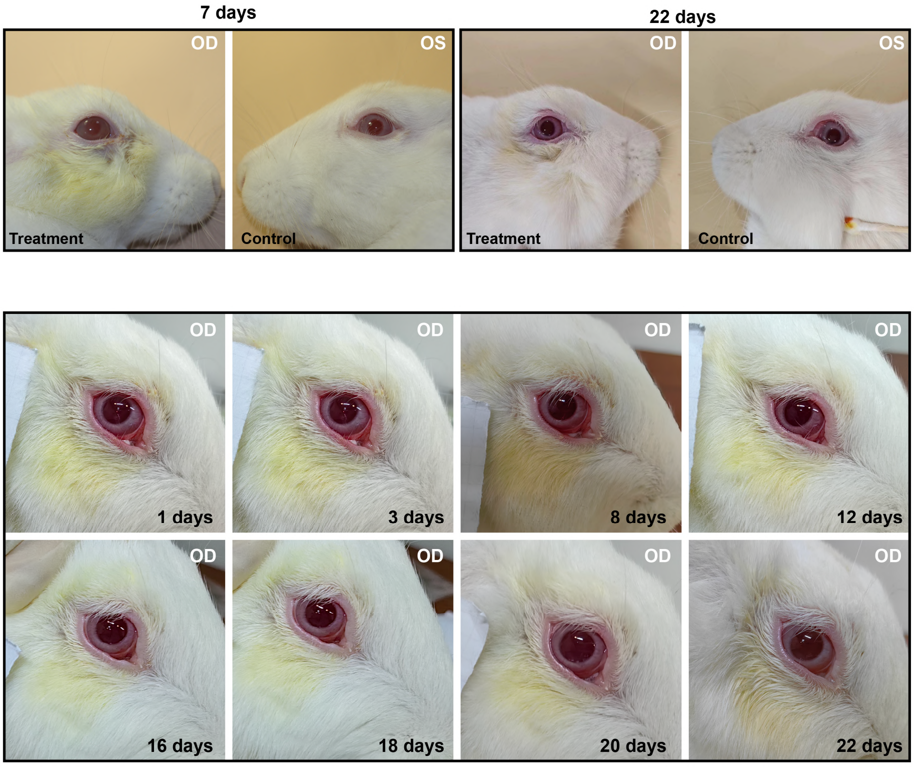

Optical imaging of the right eye (OD) and left eye (OS) 22 days post-surgery. Postoperative examination of rabbit eyes reveals a clear cornea, absence of conjunctival congestion, scleral congestion, and other surgical injuries.

Supplementary Fig. 30.

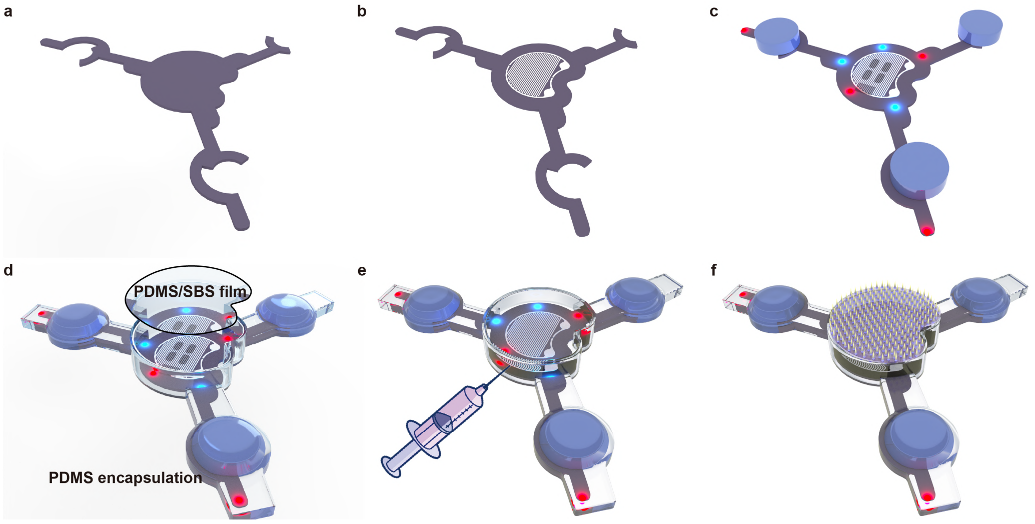

Schematic illustrations of the assembly of the myopia patch. **a** Flexible printed circuit board (FPCB) contour. **b** Interdigitated electrodes and circuit layouts. **c** Electronic components and lead zirconate titanate (PZT) soldering. **d** Encapsulation with flexible polydimethylsiloxane/polystyrene-block-polybutadiene-block-polystyrene membrane (PDMS/SBS). **e** Electrolyte solution injection. **f** Riboflavin microneedle array assembly.

Supplementary Fig. 31.

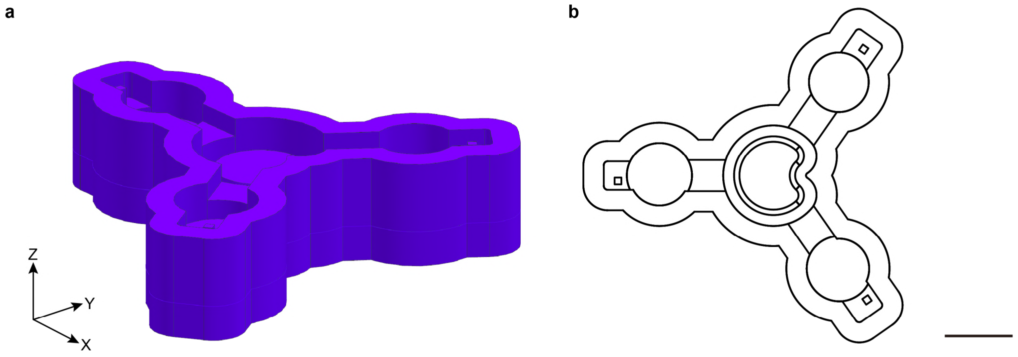

The mold of myopia patch. **a** Orthogonal views of the mold. **b** Vertical view of the mold. Scale bar, 5 mm.

Supplementary Fig. 32.

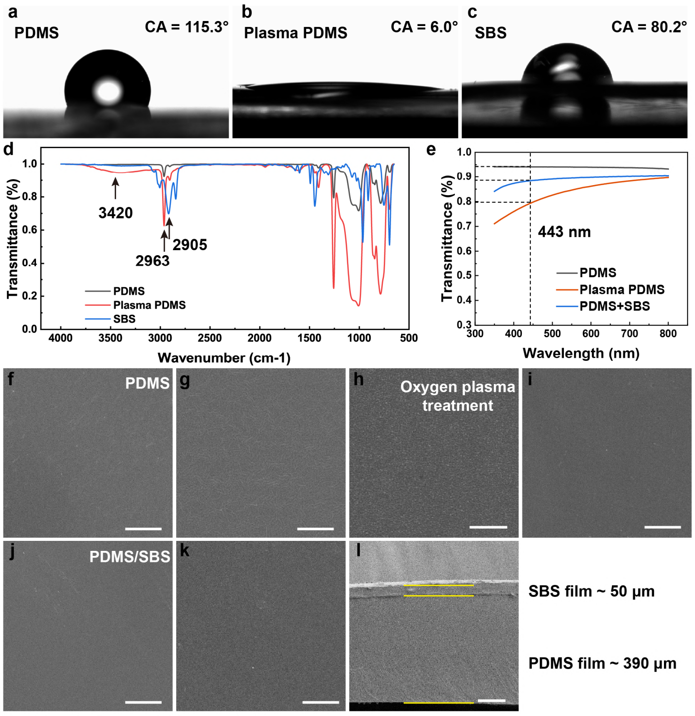

Characterization of the flexible polydimethylsiloxane/polystyrene-block-polybutadiene-block-polystyrene (PDMS/SBS) membrane. a-c Contact angles of a water droplet on (**a**) the surface of bare, (**b**) plasma-treated PDMS under oxygen gas and (**c**) SBS film. **d** Attenuated total reflection fourier transform infrared spectroscopy (ATR-FTIR) spectra for pristine PDMS, O_2 plasma modified and SBS film. **e** Transmittance for pristine PDMS, O_2 plasma modified PDMS and PDMS/SBS film. **f-g** The SEM of the PDMS film. Scale bar in (**f**): 50 μm; scale bar in (**g**): 10 μm. **h-i** The SEM of the PDMS film of oxygen plasma treatment. Scale bar in (**h**): 300 μm; scale bar in (**i**): 2 μm. **j-k** The SEM of the PDMS/SBS film. Scale bar in (**j**): 50 μm; scale bar in (**k**): 10 μm. **l** The cross-sectional image of the PDMS/SBS film. Scale bar, 100 μm. Source data are provided as a Source Data file.

Supplementary Fig. 33.

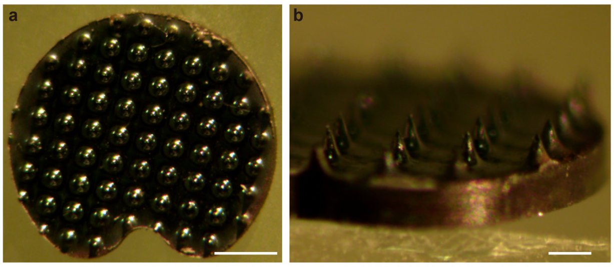

Positive mold of the microneedle array. a Vertical view of the mold. Scale bar, 2 mm. **b** Side view of the mold. Scale bar, 800 µm.

Supplementary Fig. 34.

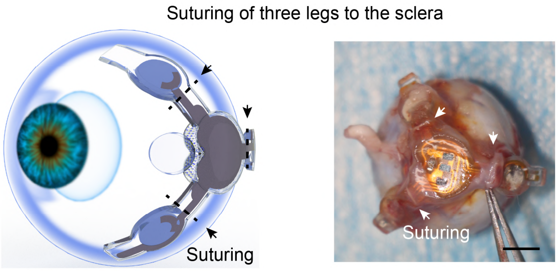

Attachment technique and location for the patch. Suturing of the patch's three legs to the sclera using a 5-0 suture. Scale bar, 3 mm.
