# Supplementary material for: A wireless battery-free eye modulation patch for high myopia therapy
Source: Nat Commun. 2024 Feb 26;15:1766. doi: 10.1038/s41467-024-46049-6 (PMC10897479; doi:10.1038/s41467-024-46049-6)
Supplement: Supplementary file 3 — Description of Additional Supplementary Files [file 41467_2024_46049_MOESM3_ESM.pdf]

Description of Additional Supplementary Files

Supplementary Movies

Supplementary Movie 1.
The deformation of the flexible membrane (mp4 format).

Supplementary Movie 2.
Real-time OCTA image of the axial length deformation in vitro porcine sclera (mp4 format).

Supplementary Movie 3.
In vivo ultrasound-induced water electrolysis in the rabbit eye (mp4 format).

Supplementary Movie 4.
In vivo ultrasound-induced collagen cross-linking process in the rabbit eye (mp4 format).
