# Supplementary material for: A wireless battery-free eye modulation patch for high myopia therapy
Source: Nat Commun. 2024 Feb 26;15:1766. doi: 10.1038/s41467-024-46049-6 (PMC10897479; doi:10.1038/s41467-024-46049-6)
Supplement: Supplementary file 8 — Reporting Summary [file 41467_2024_46049_MOESM8_ESM.pdf]

nature portfolio

Corresponding author(s): Xinyu Xue, Jie Zhong

Last updated by author(s): Feb 6, 2024

Reporting Summary

Nature Portfolio wishes to improve the reproducibility of the work that we publish. This form provides structure for consistency and transparency in reporting. For further information on Nature Portfolio policies, see our Editorial Policies and the Editorial Policy Checklist.

Statistics

For all statistical analyses, confirm that the following items are present in the figure legend, table legend, main text, or Methods section.

n/a	Confirmed	
☐	☒	The exact sample size (*n*) for each experimental group/condition, given as a discrete number and unit of measurement
☐	☒	A statement on whether measurements were taken from distinct samples or whether the same sample was measured repeatedly
☐	☒	The statistical test(s) used AND whether they are one- or two-sided<br>*Only common tests should be described solely by name; describe more complex techniques in the Methods section.*
☒	☐	A description of all covariates tested
☒	☐	A description of any assumptions or corrections, such as tests of normality and adjustment for multiple comparisons
☐	☒	A full description of the statistical parameters including central tendency (e.g. means) or other basic estimates (e.g. regression coefficient) AND variation (e.g. standard deviation) or associated estimates of uncertainty (e.g. confidence intervals)
☐	☒	For null hypothesis testing, the test statistic (e.g. *F*, *t*, *r*) with confidence intervals, effect sizes, degrees of freedom and *P* value noted<br>*Give P values as exact values whenever suitable.*
☒	☐	For Bayesian analysis, information on the choice of priors and Markov chain Monte Carlo settings
☒	☐	For hierarchical and complex designs, identification of the appropriate level for tests and full reporting of outcomes
☒	☐	Estimates of effect sizes (e.g. Cohen's *d*, Pearson's *r*), indicating how they were calculated

Our web collection on statistics for biologists contains articles on many of the points above.

Software and code

Policy information about availability of computer code

Data collection	Sweep source optical coherence tomograph and fundus angiography system (BM-400K BMizar, Toward Pi Medical Technology, Beijing, China)<br>Panoramic ophthalmoscope (Daytona (P200T), OPTOS PLC, United Kingdom)<br>Scanning Electron Microscopy (SEM, GeminiSEM 300, Germany)<br>Optical biometry (IOL Master 700, Carl Zeiss Meditec AG, Jena, Germany)<br>Ocular ultrasonography (A-B Scan, MD-2300S, China)<br>Contact Rebound Tonometer IOP mini (Icare TONOVET Plus, United State)<br>Ophthalmic camera (DX100-01A, China and DX100-01A, China)<br>Transmission Electron Microscope (JEM-1400-FLASH, Japan)<br>Digital slice scanner (Pannoramic 250, China)<br>3D Printer (ZRapid ISLA660, China)<br>Fourier Infrared Spectrometer (FT-IR) Spectrometer (INVENIO, Germany)<br>TMS-Pro Texture Analyzer was utilized (Food Technology Corporation, USA)<br>Digital Storage Oscilloscope (SDS 1202X, China)<br>KEITHLEY (DMM7510 1/2 DIGIT MULTIMETER, America)
Data analysis	Piezoelectric Voltage and Current data and mechanical test data were analyzed by using Origin 2021. Immunofluorescence data was analyzed by using Image J (Fiji) and Prism 8 (GraphPad Software). P value was calculated by Prism 8 (GraphPad Software). Sound pressure simulation was computed by using custom code in Matlab R2021a.

For manuscripts utilizing custom algorithms or software that are central to the research but not yet described in published literature, software must be made available to editors and reviewers. We strongly encourage code deposition in a community repository (e.g. GitHub). See the Nature Portfolio guidelines for submitting code & software for further information.

Data

Policy information about availability of data

All manuscripts must include a data availability statement. This statement should provide the following information, where applicable:

- Accession codes, unique identifiers, or web links for publicly available datasets
- A description of any restrictions on data availability
- For clinical datasets or third party data, please ensure that the statement adheres to our policy

Data Availability
All data supporting the findings of this study are available within the article and its supplementary files. Any additional requests for information can be directed to, and will be fulfilled by, the corresponding authors. Source data are provided with this paper. The source data is available via Zenodo at https://doi.org/10.5281/zenodo.10619661.
Code Availability
The code for data analysis and figure generation related to sound pressure simulation is available via Zenodo at https://doi.org/10.5281/zenodo.10592685.

Research involving human participants, their data, or biological material

Policy information about studies with human participants or human data. See also policy information about sex, gender (identity/presentation), and sexual orientation and race, ethnicity and racism.

Reporting on sex and gender	This study did not include human research participants.
Reporting on race, ethnicity, or other socially relevant groupings	This study did not include human samples.
Population characteristics	This study did not include human samples.
Recruitment	This study did not include human samples.
Ethics oversight	This study did not include human samples.

Note that full information on the approval of the study protocol must also be provided in the manuscript.

Field-specific reporting

Please select the one below that is the best fit for your research. If you are not sure, read the appropriate sections before making your selection.

☒ Life sciences ☐ Behavioural & social sciences ☐ Ecological, evolutionary & environmental sciences

For a reference copy of the document with all sections, see nature.com/documents/nr-reporting-summary-flat.pdf

Life sciences study design

All studies must disclose on these points even when the disclosure is negative.

Sample size	Sample size, set at n=10, was selected based on the number of biological and technical replicates deemed necessary to confidently affirm the observed effect's authenticity. This choice aimed to ensure statistically significant distinctions between experimental groups could be achieved. The determination of sample size did not involve the use of specialized statistical methods or pre-calculated estimations. The chosen sample size was considered adequate to provide robust and reliable results for the study's objectives.
Data exclusions	No data were excluded from analysis.
Replication	All our computation results are replicable.
Randomization	For all subjects, the left eye served as a blank control, while the right eye functioned as a control group before the experiment and as the experimental group afterwards.
Blinding	Our study does not involve group allocation that requires blinding.

Reporting for specific materials, systems and methods

We require information from authors about some types of materials, experimental systems and methods used in many studies. Here, indicate whether each material, system or method listed is relevant to your study. If you are not sure if a list item applies to your research, read the appropriate section before selecting a response.

Materials & experimental systems

n/a	Involved in the study
☐	☒ Antibodies
☒	☐ Eukaryotic cell lines
☒	☐ Palaeontology and archaeology
☐	☒ Animals and other organisms
☒	☐ Clinical data
☒	☐ Dual use research of concern
☒	☐ Plants

Methods

n/a	Involved in the study
☒	☐ ChIP-seq
☒	☐ Flow cytometry
☒	☐ MRI-based neuroimaging

Antibodies

Antibodies used	Anti- IBA 1 Mouse mAb (GB12105; Servicebio, China; diluted: 1:100 with the primary antibody dilution buffer (G2025, Servicebio))<br>Cy3 conjugated Goat Anti-mouse IgG (H+L)(GB21301; Servicebio, China; diluted: 1:100 in PBS)<br>Anti- GFAP Rabbit pAb (GB11096, Servicebio, China; diluted: 1:100 with the primary antibody dilution buffer (G2025, Servicebio))<br>fluorescein isothiocyanate (FITC)–conjugated goat anti-rabbit IgG secondary antibody (GB22303, Servicebio, China; diluted: 1:100 in PBS)<br>TMR (Red) Tunel Cell Apoptosis Detection Kit (Servicebio, G1502-50T;Recombinant TdT enzyme TMR-5-dUTP Labeling Mix Equilibration Buffer (1 5 50) volume ratio)
Validation	All antibodies used in this study are commercially available and validated by the vendor for the species and assay. Specific validation information is available on the website from the vendors and listed below:<br>Anti- IBA 1 Mouse mAb, https://www.servicebio.cn/goodsdetail?id=603<br>Anti- GFAP Rabbit pAb, https://www.servicebio.cn/goodsdetail?id=1376<br>TMR (Red) Tunel Cell Apoptosis Detection Kit (Servicebio, G1502-50T), https://www.servicebio.cn/search-result?search=G1502-50T

Animals and other research organisms

Policy information about studies involving animals; ARRIVE guidelines recommended for reporting animal research, and Sex and Gender in Research

Laboratory animals	General grade New Zealand three-month-old white rabbit weighing 2.5-3.0 kg were treated in accordance with the approved study protocols and relevant regulations. All experimental rabbits were purchased from the Biotechnology Corporation of Dashuo (Chengdu, China). The rabbits were maintained under 12-h light/12-h dark conditions. Rabbits with any form of lesion in the cornea, lens or fundus were excluded.
Wild animals	No wild animals were used in this study.
Reporting on sex	Both sex rabbit were used in this study indiscriminately.
Field-collected samples	No field collected samples were used in this study.
Ethics oversight	These studies adhered to the ARVO Statement for the Use of Animals in Ophthalmic and Vision Research and were approved by the Animal Care and Use Committee of Sichuan Provincial People's Hospital.

Note that full information on the approval of the study protocol must also be provided in the manuscript.

Plants

Seed stocks	*Report on the source of all seed stocks or other plant material used. If applicable, state the seed stock centre and catalogue number. If plant specimens were collected from the field, describe the collection location, date and sampling procedures.*
Novel plant genotypes	*Describe the methods by which all novel plant genotypes were produced. This includes those generated by transgenic approaches, gene editing, chemical/radiation-based mutagenesis and hybridization. For transgenic lines, describe the transformation method, the number of independent lines analyzed and the generation upon which experiments were performed. For gene-edited lines, describe the editor used, the endogenous sequence targeted for editing, the targeting guide RNA sequence (if applicable) and how the editor was applied.*
Authentication	*Describe any authentication procedures for each seed stock used or novel genotype generated. Describe any experiments used to assess the effect of a mutation and, where applicable, how potential secondary effects (e.g. second site T-DNA insertions, mosiacism, off-target gene editing) were examined.*
